# The Development of Novel Therapies for the Treatment of Acute Myeloid Leukemia (AML)

**DOI:** 10.3390/cancers4041161

**Published:** 2012-11-02

**Authors:** Sarit Assouline, Eftihia Cocolakis, Katherine L. B. Borden

**Affiliations:** 1 Department of Medicine and Oncology, Segal Cancer Centre, Jewish General Hospital, Montreal, QC, H3T 1E2, Canada; E-Mail: ecocolakis@jgh.mcgill.ca; 2 Department of Pathology and Cell Biology, Institute of Research in Immunology and Cancer (IRIC), Université de Montréal, Pavillion Marcelle-Coutu, Chemin Polytechnique, Montreal, QC, H3T 1J4, Canada

**Keywords:** acute myeloid leukemia, targeted therapies, early phase trials

## Abstract

Acute myeloid leukemia (AML) is nearly always a fatal malignancy. For the past 40 years, the standard of care remains a combination of cytarabine and an anthracycline known as 7 + 3. This treatment regimen is troubled by both low survival rates (10% at 5 years) and deaths due to toxicity. Substantial new laboratory findings over the past decade have identified many cellular pathways that contribute to leukemogenesis. These studies have led to the development of novel agents designed to target these pathways. Here we discuss the molecular underpinnings and clinical benefits of these novel treatment strategies. Most importantly these studies demonstrate that clinical response is best achieved by stratifying each patient based on a detailed understanding of their molecular abnormalities.

## 1. Introduction

Traditionally, therapies for AML have relied on the use of non-targeted cytotoxic agents, which are often not well tolerated and leave much room for improvement in terms of clinical response. The greatest strides in treating myeloid malignancies have come from the development of targeted agents. These include all *trans*-retinoic acid and arsenic trioxide in acute promyelocytic leukemia, which overcome the differentiation block at the promyelocyte stage by targeting the retinoic acid receptor-associated translocation [1]. Similarly, imatinib mesylate and second and third generation tyrosine kinase inhibitors (TKI) have drastically changed the outlook for patients with chronic myeloid leukemia, by inhibiting the constitutively active bcr-abl protein [2].

Other than these few therapeutic successes, there have been few clinical advances in the treatment of myeloid malignancies, and in particular AML. The standard treatment option for the majority of young, fit patients with AML remains continuous infusion of cytarabine for days 1 to 7 and an anthracycline, daunorubicin or idarubicin, as a daily bolus for days 1 to 3 (7 + 3), a long-lived standard combination in use for at least the last 40 years [3]. However, one recent noteworthy development has been the observation based on two large intergroup studies [4,5] that an increased dose of daunorubicin is associated with improved survival. In the ECOG study, which focused on younger patients with AML, this benefit was restricted to those with favorable and intermediate cytogenetics [4]. In the HOVON study, which tested increased doses of daunorubicin in patients aged 60 and older, higher response rates and overall survival was observed in patients aged 60 to 65 and not older, as well as among patients with core-binding factor leukemias [5]. Still, the median overall survival at 2 years ranges from 38 to 50% for patients receiving higher dose daunorubicin, depending on the age group [4,5].

Many elderly patients, who comprise more than half of those with AML, are unfit for any induction chemotherapy or likely not to benefit due to poor cytogenetic risk [3]. For these patients, the only therapy of demonstrated benefit in the setting of a Phase III trial is low-dose cytarabine. In the Medical Research Council study by Burnett and colleagues, this treatment was associated with a complete response (CR) rate of 18% and median survival of 80 weeks [6]. Given the low response rates and median survival, this standard is not universally accepted or employed [7].

Novel targeted therapies are needed to improve survival for more patients with AML. Numerous agents are in development with the aim of targeting both well established and more recently identified leukemogenic pathways. This review begins with a discussion of some promising novel targeted agents recently tested in AML patients and, within this context, the clinical hurdles to drug development in AML are highlighted. Non-targeted, conventional chemotherapeutic agents and combinations of these agents remain outside the scope of this review.

## 2. Molecular Targets of New Drug Development in AML

### 2.1. Gene Mutations

While the development of novel therapies has been slow, the knowledge of mutations associated with AML has increased dramatically in recent years. There are known translocations e.g., t(15;17), t(8;21), inv 16, t(16;16) which are associated with a favorable prognosis; e.g., t(15;17), t(8;21), inv 16, t(16;16), the so-called core binding factor (CBF) leukemias [8,9]. It is also well established that a complex karyotype (three or more cytogenetic abnormalities) [8,9] and a monosomal karyotype are associated with a poor outcome [10,11]. Finally, numerous, recurrent genetic mutations have been shown to predict for survival in patients with cytogenetically normal AML treated with 7 + 3 [12]. In one large ECOG study, 97.3% of AML patients had at least one identifiable mutation (that is, one occurring in greater than 5% of patients), regardless of the presence of cytogenetic abnormalities [13]. A detailed algorithm was devised to predict survival after 7 + 3 based on the combination of the expression of seven genes, including the well known internal tandem duplication of FLT3 and mutations of nucleophosmin (NPM1). Furthermore, survival was improved among patients with DNA methyl transferase 3A (DNMT3A) or NPM1 mutations or mixed lineage leukemia (MLL) translocations when higher doses of daunorubicin were employed [13]. Subdividing patients who can benefit from higher doses of chemotherapy on the basis of gene mutations is a step in the direction of personalized therapy for AML, but still does not make full use of the clearly identified molecular heterogeneity of AML.

Mechanisms of leukemogenesis have been attributed to many of the known mutations, but most have not been the subject of a targeted therapeutic plan. This may be due to: (1) a lack of identified therapies to target the mutations; (2) the genetic complexity of AML which is often characterized by several co-existing mutations that all contribute to leukemogenesis; and, (3) low numbers of patients with any given mutation, which makes it difficult to devise a clinical trial with a reasonable accrual rate. For example, c-kit mutations have been described in CBF leukemias [14,15]. However, despite the presence of c-kit inhibitors, results with these have never been reported, perhaps because this subgroup represents a relatively small number of patients. However, given the frequency of FLT3-ITD mutations in AML and the existence of inhibitors, this gene mutation has been targeted.The exception, of course, includes therapies targeting FLT3-ITD.

The most promising target for AML therapy in this past decade has been the FLT3 (FMS-like tyrosine kinase 3) protein. FLT3 is a transmembrane receptor tyrosine kinase that belongs to the same family as FMS, kit, and PDGFRa/b. It is mutated in about 30% of all AML [16]. The mutations include in-frame internal tandem duplication (ITD) of the transmembrane domain in 95% of cases, and a tyrosine kinase domain (TKD) mutation at aspartic acid residue 835 in the remainder, with other mutations rarely observed [17]. For AML patients with normal cytogenetics, the presence of an FLT3 ITD mutation, is associated with poorer progression-free and overall survival [12].

In AML, there is constitutive activation of FLT3 either due to interference with the negative regulatory function of the juxtamembrane region with ITD mutations, or changes in the activation loop with TKD mutations [18]. As a result, there is autophosphorylation and direct or indirect phosphorylation of several proteins that, in turn, activate the PI-3-kinase/AKT, RAS/MAPK, and STAT5 pathways, ultimately inducing cellular proliferation and inhibiting apoptosis [19]. Interestingly, isolation of FLT3-mutated human CD34+CD38− leukemia stem cells indicated that all had the FLT3 mutation, and, injection of these cells into NOD/SCID mice resulted in leukemogenesis that was entirely FLT3 mutated [20]. Thus, FLT3 mutations can drive leukemogenesis. Clinical evidence suggests that the FLT3 mutation may occur in a sub-clone of the leukemia stem cell (LSC), including the finding that in the context of relapsed AML, there is loss of the ITD mutation 16% of the time and loss of the TKD mutation 50% of the time [21,22].

Given these data and the availability of several TKIs with activity against FLT3-mutated leukemia [23], several clinical trials have been undertaken. There are multi-targeted TKI with activity against FLT3, including lestaurtinib, midostaurin and sorafenib; and, more specific inhibitors of FLT3, quizartinib and tandutinib. As single agents, all exhibit some anti-leukemia activity, but the development of tandutibin has been limited by its toxicity profile [23]. The multi-targeted agents have activity in AML with wild-type FLT3, but activity is greater in the presence of FLT3 ITD or TKD (Table 1).

**Table 1:** Representative single agent activity of targeted therapies in AML.

Treatment	Complete response %	Partial response %	Blast response %	Comments	Ref.
**FLT3 inhibitors**					
Sorafenib n = 50; n = 39 with FLT3 ITD or D835 point mutation or both	10% (13% FLT3 mutation)		34% (all with FLT ITD)	Phase I, relapsed/refractory AML	[24]
Midostaurin n = 95; n = 35 FLT mutant	0	1.6%	71% FLT3 ITD 42% FLT3 WT	Phase II, relapsed/refractory MDS and AML	[25]
Lestaurtinib n = 14	0%	0%	29%	Phase II, relapsed/refractory AML with FLT3 ITD	[26]
Lestaurtinib n = 5 with FLT 3 mutation n = 22 WT	0	0	60% (FLT3 mutated) 23% (WT)	Phase II, newly diagnosed AML in the elderly	[27]
AC220 n = 76; n = 47 with FLT3 mutant	12% (22% flt3 mutated; 6% WT; 18%unk)	18% (33% flt3 mutated; 13% WT; 18%unk)		Phase I, relapsed refractory AML unselected for FLT3 ITD	[28]
**Epigentic modulation**					
5-azacytidine n = 113	18% (*vs*. 16% in BSC)			Subanalysis of patients with low blast count in phase III trial	[29]
Decitabine n = 485	17.8% (*vs*. 7.8% conventional care)			Phase III trial compared to conventional care (TC) AML	[30]
**mRNA processing and translation**					
Ribavirin n = 11	7%	13%	27%	AML FAB M4 and M5 M4 and M5 subtypes, phase II, refractory, relapsed or newly diagnosed, unfit for induction chemotherapy	[31]
**PI3K/Akt/mTOR **					
Deformolus (Rapamycin analogue) n = 22	0%	0%	0%	Phase II	[32]
**Protein recycling**					
Tosedostat n = 73	12%	10%		Phase II, patients aged 60 and over with relapsed, refractory disease	[33]
**CD33**					
Gemtuzumab ozogamycin n = 142	29%		17%	Phase II; relapsed AML; complete response includes patients with incomplete platelet recovery	[34]
**Farnesylation and RAS targeting**					
Farnesyl Transferase Inhibitor R115777 n = 34	6%	24%		Phase II; relapsed/refractory AML	[35]

WT = wild type; unk = unknown mutational status; BSC = best supportive care.

There have been several reasons identified for the limited success of FLT3 inhibitors. The problem of bioavailability has been highlighted by a randomized Phase III trial of induction chemotherapy combined with lestaurtinib in relapsed AML with FLT3-ITD, where there was no improvement in survival over chemotherapy alone. This was attributed to decreasing plasma levels of lestaurtinib with time, and increasing alpha-1 acid glycoprotein, which binds lestaurtinib *in vivo* [36]. In addition, *in vitro* studies indicate that samples with high FLT3-ITD allele burden are more sensitive to the cytotoxic effect of FLT3 inhibitors compared to samples with a low allele burden [21,37]. FLT3 upregulation has also been observed *in vivo* following exposure to lestaurtinib [27]. Finally, recent compelling data indicate that immunosuppresive therapy, including chemotherapy, increases circulating levels of FLT3 ligand, which may reduce the ability of TKIs to inhibit FLT3 activity [38]. Nonetheless, there is an ongoing Phase III clinical trial testing midostaurin with induction chemotherapy in newly diagnosed AML with FLT3-ITD (clinicaltrials.gov NCT00651261). If results of this trial are positive, they will dramatically change the treatment of newly diagnosed AML with FLT3 mutation. It may be that using these agents in the frontline setting will allow for greater efficacy.

### 2.2. Epigenetic Changes

Anomalous epigenetic changes, including DNA hypermethylation and histone acetylation/deacetylation occur frequently in acute leukemia. The recurring chromosomal translocations seen in AML lead to the generation of chimeric fusion oncoproteins e.g., TEL-AML, AML-ETO, PML-RARa, *etc*. In many cases these fusion proteins contribute to the development of leukemia partly by disrupting the modification of chromatin, through recruitment of chromatin-modifying coregulators [39]. In AML, aberrant CpG island hypermethylation of tumor suppressor genes leads to transcriptional shut-down and involves the recruitment of methyl-binding proteins and histone deacetylases (HDACs) to regions near transcription start sites [40,41]. Unlike chromosomal deletions that lead to an irreversible loss of function, transcriptional repression by epigenetic mechanisms such as histone deacetylation and promoter DNA methylation can be reversed using HDAC inhibitors (HDACi) and hypomethylating agents, respectively. These compounds are effective at controlling leukemic cell growth in the laboratory. The cytidine analogs 5-aza-2'-deoxycytidine (decitabine) and 5-azacytidine can reactivate tumor suppressor genes silenced by promoter hypermethylation [42]. Incorporation of these agents into DNA leads to subsequent DNA demethylation via their irreversible inhibition of DNMTs. Importantly, many of these compounds have other functions as well, e.g., azacytidine is incorporated into nearly all forms of RNA [43,44,45], and the contribution of these has not been well studied.

Clinically, 5-azacytidine treatment leads to improved overall survival in AML patients with low blast counts, as demonstrated in a sub-analysis of the AZA-001 study of high risk myelodysplastic syndrome, in which approximately one third of patients had low blast count AML [30]. When decitabine, a 5-azacytidine pro-drug, was compared to best supportive care in a Phase III trial of AML, there was no survival benefit in patients receiving decitabine over the control arm at the planned analysis point. But, the data were analyzed one year later, at which point a small survival advantage of 2 months became statistically significant (*p* = 0.037). A reason for the modest benefit was that patients with high percentage blast count and high white counts were included in this study. This was in contrast to the patients enrolled in the AZA-001 study, who had low white counts and blast counts not exceeding 30% [31]. Finally, unique subtypes of AML have been identified on the basis of DNA methylation signatures, which were found to be predictive of survival but not yet of response to hypomethylating agents [46]. HDACi have been tested in AML with modest results [47]. Combinations of HDACi with DNA hypomethylating agents have been reported [48,49] or are underway. Such combinations are appealing, as these epigenetic modes of regulation cooperate. However, so far, they have not yielded high response rates.

### 2.3. mRNA Processing and Translation

Messenger RNA (mRNA) processing and mRNA translation are important steps in the regulation of protein levels in cells and these processes are coupled to essential cellular events such as growth, proliferation, differentiation and apoptosis. In normal hematopoietic cells, translation of transcripts into protein is tightly regulated at the initiation phase by signal transduction pathways including PI3K/AKT pathways that can ultimately affect eukaryotic translation initiation factor 4E (eIF4E). Most AMLs are characterized by elevated PI3K and Akt activity [50,51]. Further, eIF4E levels themselves become highly elevated in the M4 and M5 subset of AML at both the RNA and protein levels [32,52,53,54]. Additionally, eIF4E is highly concentrated in the cell nucleus in these cells [32,52,53,54]. Elevated eIF4E levels and activity increase translational initiation for a subset of mRNAs and thereby enable multiple ribosomes to translate the same transcript simultaneously. In this way, eIF4E increases translational efficiency of a specific subset of transcripts that are associated with proliferation and survival signaling. Further, eIF4E enhances the mRNA export of a subset of transcripts also involved in proliferation and survival which increases their cytoplasmic levels and thus their availability to the translation machinery. Both of these activities are stimulated by the phosphorylation of eIF4E which occurs via Mnk kinase [55,56,57,58,59]. Thus, targeting PI3K, Akt, Mnk and eIF4E are all reasonable strategies to target these processes.Cancer cells appear to develop an oncogene addiction to eIF4E thereby providing a therapeutic window for targeting this protein [32,60,61].

The only direct approach to target eIF4E in AML patients in the clinic thus far has been with ribavirin [52,32,60,62]. Ribavirin is a well-characterized broad spectrum nucleoside analogue with antiviral activity against a range of viruses [52]. Ribavirin can act as competitive inhibitor of the natural ligand of eIF4E, the methyl 7 guanosine cap. Consistently, ribavirin targets eIF4E directly in a variety of systems, thereby inhibiting translation and/or mRNA export of sensitive transcripts. A small phase II clinical trial examining the efficacy of ribavirin treatment in refractory, relapsed or unfit for chemotherapy M4 and M5 AML patients demonstrated clinical activity and associated molecular responses. Out of 11 patients, three achieved a partial or complete response and three had blast responses [32]. Common leukemia drugs such as ara-C and idarubicin combine with ribavirin to further reduce colony number in primary patient specimens *ex vivo* [53] and served as the starting point for a phase I trial in M4/M5 AML with patients using ribavirin in combination with low dose ara-C (ClinicalTrials.gov NCT01056523) [63].

### 2.4. PI3/AKT/mTOR Pathways

Most AML cells show activated PI3K/AKT/mTOR pathways [50,51]. For example, the Carroll laboratory showed that PI3 kinase inhibitors such as LY294002, or the more specific inhibitor PI-103, induced cell death in primary AML cells *ex vivo* [64,65]. Akt activation assays confirmed that Akt activity was high in most specimens. Inhibition of Akt with perfosine, which affects other kinases as well [66], in preclinical studies suggested that combining this with MEK inhibitors kill AML cells *ex vivo* [67]. There is an ongoing clinical trial to test this hypothesis using perifosine and UCN-1 (Clinicaltrials.gov NCT00301938).

Downstream of PI3K and AKT is the mTOR pathway. mTOR activation results in the phosphorylation of both eIF4E binding protein BP1 and ribosomal S6 protein. In AML, S6 phosphorylation is a robust marker (see below). Early studies with rapamycin, an mTORC1 inhibitor, failed to show responses in clinical trial as a single agent [68]. However, it may play a role as a chemosensitizing agent [69]. Combinations of chemotherapy with PI3K and TORC1/2 inhibitors could achieve clinical responses superior to either agent alone [33,70]. Supporting this idea, the combination of one rapalogue, temsirolimus, with clofarabine in elderly high risk AML patients showed an overall response rate of 21% which was comparable to single agent clofarabine [71]. However, there was a much more substantial 75% response rate in the 12 patients that demonstrated inhibition of S6 phosphorylation [71]. One of the most robust markers of mTOR signaling in AML is phosphorylation of the S6 protein which is readily observed by flow cytometry in primary patient specimens and thus can be used as a molecular marker for drugs that target this pathway [36]. Interestingly, less than 50% of cells show S6 activation at any given time in primary AML specimens *ex vivo* [36]. This heterogeneity could arise due to differences in drivers of oncogenesis and/or differences related to cell cycle status. In a sirolimus plus chemotherapy trial of refractory and relapsed AML patients, again there was a striking correlation between ribosomal S6 phosphorylation and response [72].

### 2.5. Protein Recycling

Tosedostat is an inhibitor of aminopeptidase and therefore acts by depleting amino acids. In the HL-60 leukemia cell line, gene array studies have shown that this amino acid depletion resembles an amino acid deprivation response (AADR) [73]. In the same cell line model, tosedostat also inhibits phosphorylation of mTOR substrates and reduces protein levels, both of which are indicative of amino acid depletion, and leads to increased concentrations of intracellular small peptides [73]. By blocking protein recycling, tosedostat likely depletes sensitive tumor cells of amino acids thereby generating an antiproliferative effect.

In a phase II study of tosedostat as a single agent in elderly patients with relapsed AML, median age 72, and half with refractory disease prior to starting therapy, 12% had a complete response and 10% a partial response. Some of these responses occurred among patients having failed hypomethylating agents and patients who had MDS previously. The median survival among patients with a CR was 323 days, 195 days among patients with partial response, and 162 days in those with stable disease [34]. Based on these promising results, a phase III study is planned in patients with high risk MDS and AML who have failed therapy with a hypomethylating agent.

### 2.6. Monoclonal Antibody Therapy against CD33: Gemtuzumab Ozogomycin

Monoclonal antibody therapy has proven extremely successful in other malignancies. In AML, gemtuzumab ozogomycin (GO) is an antibody-drug conjugate where a humanized monoclonal IgG4 antibody directed against CD33 is linked to a derivative of calicheamicin, a potent DNA-binding cytotoxic antibiotic [74]. CD33 antigen is expressed on leukemic myeloblasts in approximately 90% of patients, making GO an attractive anti-leukemic therapy [75]. In fact, GO was granted approval by the FDA in 2000 for the treatment of relapsed CD33-positive AML patients that were not considered candidates for cytotoxic chemotherapy and were over the age of 60 based on three open label trails showing a 30% complete response rate and favorable safety profile [35,76]. However, results from Phase III combination trials have provided mixed outcomes in first-line AML patients. The FDA required, post-approval SWOG study (S0106) was terminated early after an interim analysis revealed a significantly higher risk of fatal induction adverse events (5.8% *vs*. 0.8%) and no improvement in complete remission rates, relapse free survival, post-consolidation disease free survival, or overall survival. Untreated AML patients under the age of 61 were randomized to cytarabine and daunorubicin with or without GO (6 mg/m^2^) [77]. In June 2010, GO was withdrawn from the market because of concerns about safety and lack of efficacy [78].

AML15 compared the addition of GO to one of three induction schedules and/or one of two consolidation schedules [79]. A total of 1,113 AML patients between the ages of 15 and 60 years were randomly assigned to receive GO as part of their induction therapy (daunorubicin and cytarabine; cytarabine, daunorubicin, and etoposide; or fludarabine, cytarabine, granulocyte colony-stimulating factor, and idarubicin) and a total of 948 AML patients of the same age group were randomized to GO with or without consolidation therapy (amsacrine, cytarabine, and etoposide or high-dose cytarabine). There was no significant increase in toxicity noted in the patients on the GO arms. Unfortunately, there was no difference in response and overall survival when GO was added to either induction or consolidation therapy. However, a subset of patients with favorable cytogenetics that received GO as part of their induction regimen showed a significant survival benefit [79]. AML16 examined the addition of GO to induction chemotherapy in patients over the age of 60 [80]. In this case, there was a small improvement in overall survival with the addition of GO. Furthermore, a meta-analysis of both trials indicated a significant improvement in survival overall [80].

Finally, a phase III study by Castaigne and colleagues performed in 26 centers in France, evaluated the benefit of low dose GO when added to 7 + 3 in newly diagnosed AML patients between the ages of 50 and 70 [81]. A total of 280 patients were randomly assigned to either 7 + 3 with or without GO at 3 mg/m^2^. GO was given on day 1, 4 and 7 in order to deliver a cumulative dose of 9 mg/m^2^ and to reduce liver toxicity. Event free survival at 2 years showed a significant advantage at 41.4% in patients treated with GO in comparison to 15.6% for the chemotherapy alone arm. Similarly, there was an overall survival benefit of 53.2% *versus* 41.9% as well as a benefit in relapse free survival of 50.3% *versus* 22.7% in the GO-containing regimen, and no increase in treatment related death [81]. The findings from this trial should spark new interest in GO as a front-line therapy for AML.

## 3. Phase II Clinical Trial Design Strategies for New Drug Development in AML

New drug development relies heavily on the results of Phase II trials, which determine whether a therapy will be tested in large, Phase III trials. Phase II clinical trial design includes not only the method by which patients will be enrolled and the sample size, but also the dose and schedule for single and combination drugs, patient selection, determination of primary and secondary endpoints, and response assessments. This section reviews some of these elements and current difficulties.

### 3.1. Clinical Trial Design

Most phase II trials in AML are single arm, uncontrolled trials with small sample sizes [82] mainly because AML is a relatively rare disease. In the absence of a comparator arm, it is uncertain whether the experimental therapy increases response rates over standard chemotherapy or no therapy at all, this has been a criticism of many AML trials [82]. Having a standard of care as the control arm within the phase II trial design could make such studies more robust.

In order to deal with the small sample sizes and lack of comparator arm, the “Pick-a-Winner” design has been proposed [83]. In this design, patients with AML are enrolled and randomized either to a standard of care, or one of several experimental therapies. These experimental therapies can be added or removed over the life of the trial based on a pre-specified response rate. In this way, novel therapies are compared to a standard, and ineffective therapies can be rejected quickly [83]. However, because of the molecular and clinical heterogeneity of AML, as discussed, this process may erroneously reject an effective therapy. Furthermore, this design assumes access to numerous anti-leukemia therapies which is not always feasible.

### 3.2. Designing Combination Agent Trials

Single agent studies are obviously limited by both low response rates and remission duration. An obvious way to circumvent these problems is to combine them with standard chemotherapeutic drugs known to have activity in AML and, ideally, synergy *in vitro* with the novel agent. A recent example includes the combination of salvage chemotherapy and plerixafor, an inhibitor of CXCR4, in relapsed and refractory AML [84]. CXCR4 is a cell membrane receptor found on AML blasts and marrow stem cells. It provides a survival signal to these cells when hybridized with its ligand CXCL12, which is produced by marrow stromal cells. Plerixafor is used for stem cell mobilization by inhibiting the interaction between marrow stroma and stem cell [85,86]. In AML, inhibiting the function of CXCR4 may enhance chemosensitivity by removing the AML blast from its protective environment and also by inhibiting survival signaling through CXCR4 [87]. In mouse models of AML, plerixafor enhances response rates to chemotherapy [87]. On the basis of these data, a phase I/II study was completed in relapsed/refractory patients with AML given mitoxantrone, etoposide and cytarabine (MEC) chemotherapy and plerixafor. Plerixafor successfully mobilized AML blasts, as reported in the study, and the therapy appeared to be safe, not inducing hyperleucocytosis or profound aplasia [84].

In combining targeted agents with chemotherapeutic drugs, toxicity becomes a concern. In the SWOG study of GO and induction chemotherapy, there was excess death in the GO arm attributed to the dose and schedule of GO, whose toxicity may have been enhanced by chemotherapy [77]. When the dosing schedule of GO was changed to deliver lower doses but to maintain an adequate cumulative dose, an advantage was observed in terms of overall survival [81]. In the lestaurtinib plus chemotherapy trial, the sub-optimal pharmacokinetics of lestaurtinib and the upregulation of FLT3 ligand by chemotherapy likely contributed to the negative results [36]. Finally, when examining the serum steady state levels of ribavirin in combination with low dose cytarabine, they were lower among patients given cytarabine compared to those given ribavirin alone leading to dose escalation of ribavirin in order to achieve appropriate plasma levels, and this was associated with clinical responses including remissions [63]. As can be seen, combining novel agents with chemotherapy in AML is challenging and requires consideration of altered pharmacokinetics, which can affect both toxicity and response rates.

One sophisticated approach to ensuring greater predictability in drug delivery is exemplified by the development of CPX-351, a liposomal preparation of cytarabine and daunorubicin, which maintains a fixed ratio of 5:1 cytarabine:daunorubicin. This preparation has shown promise in a small randomized Phase II trial of older patients with newly diagnosed AML. By maintaining a fixed ratio of drugs, CPX-351 maximizes synergy and minimizes antagonism *in vitro*. In clinical trials, the fixed ratio is maintained [88], and in Phase II testing, in comparison to standard 7 + 3, CPX-351 induces more complete responses, and is associated with less induction mortality and an improvement in overall survival [89]. How this technology can be applied more generally remains to be seen.

### 3.3. Impact of Patient Factors in Clinical Trial Design

An important consideration in the development of new therapies for AML is the selection of patients for Phase I and II trials. For the majority of trials, these are patients with relapsed AML or elderly patients for whom induction chemotherapy is not feasible. These are important populations to target given the dearth of therapeutic options. However, these patients are often more frail, less able to tolerate chemotherapy and have a more resistant and more genetically heterogeneous disease than young, newly diagnosed patients.

From our experience with Phase I and II trials in AML, the early attrition rate due to rapid disease progression, infection, or overall clinical deterioration approaches 33%. Such a high rate of early attrition could result in a therapy being falsely determined to be ineffective if all patients having taken at least one dose of treatment are considered evaluable for response.

Further, difficulties in testing new drugs in refractory and relapsed AML patients include the development of drug resistance mechanisms (including *p*-glycoprotein and other mechanisms) that affect not only standard agents, but also the novel therapeutics. For instance, if drug transporters are downregulated as a response to one therapy, all therapies requiring this drug transporter, despite being based on different intracellular pathways, would be expected to fail. This leads to a conundrum in using heavily pretreated patients in these studies as drugs effective as first line agents may fail in this setting.

Another common issue in clinical trials of agents without a rapid rate of response, which is the case for many novel agents, has been that patients with a high peripheral blood blast count, or rapidly doubling white count are less likely to respond [31]. Some groups have used the strategy of selecting patients with leucopenia and slow white count doubling time for their trials in order to allow time for therapies to work [34,90]. Alternatively, combinations with more aggressive chemotherapy can help to decrease blast counts quickly to allow the targeted agent time to act on the remaining leukemic cells.

### 3.4. Using Molecular Correlates to Design Clinical Trials

As with other cancers, a detailed understanding of the molecular profile of a particular AML is needed to appropriately place individuals on suitable clinical trials. Mutational testing is a first step in identifying potential targets and in selecting therapies, but, as can be seen from the FLT 3 inhibitor data, targeting a single mutation is likely insufficient. In addition, other molecular predictors of response characterize certain AMLs, including eIF4E overexpression in AML FAB subtypes M4 and M5. In the proof-of-principle trial, responses to ribavirin were seen irrespective of FLT3 mutation status [32]. As discussed, S6 phosphorylation is a predictor of response to mTOR inhibitors [71,72]. Finally, gene signatures, as determined by gene expression profiling, can predict for response to certain therapies. An example includes the combination of tipifarnib and etoposide. Tipifarnib alone has limited activity in elderly patients with newly diagnosed AML [91], however when combined with etoposide, response rates are higher and, more importantly, patients with a particular two gene signature have a significantly higher response rate [90]. Added to that, data indicate that adequate inhibition of target is associated with a better response rate [36]. This suggests that studies should not only assess target inhibition but should respond to inadequate inhibition with intrapatient dose escalation, toxicity permitting.

## 4. Conclusions

Some of the most relevant pathways and mutations in AML have been outlined, as well as the therapies being developed to target these pathways. No single molecular abnormality accounts for leukemogenesis in AML and given this complexity, it is not surprising that targeted agents have not led to widely applicable therapies. However, some of these agents, even as monotherapies have been associated with activity including some complete responses, when the most apt patient cohorts are considered (Table 1). How best, then, to utilize the plethora of both laboratory and clinical information to enhance the effectiveness of AML therapies in the future? Rejecting any given therapy based on a lack of overall benefit in Phase III testing is too drastic an approach in the context of a disease where there is so much molecular complexity, and where therapies likely to have an impact will benefit only specific subgroups of patients as has been demonstrated by several examples provided here. Along the same vein, allowing therapies to enter Phase III testing, where hundreds of patients are to be enrolled, without refining the patient selection process based on the likelihood of responding, seems not only wasteful of our acquired collective knowledge, but, more importantly, of our patients’ precious time and wellbeing. While this may affect the rate of accrual, particular attention to patient selection for trial entry, based on molecular variables, in particular, but also on clinical variables, can make the difference between identification to a therapy which can provide benefit to some patients with AML rather than no benefit to anyone.
